# Effect of Nutrient Addition on the Productivity and Species Richness of Grassland Along With an Elevational Gradient in Tajikistan

**DOI:** 10.3389/fpls.2021.765077

**Published:** 2021-12-10

**Authors:** Lian-Lian Fan, Okhonniyozov Mekrovar, Yao-Ming Li, Kai-Hui Li, Xue-Xi Ma, Jie-Fei Mao

**Affiliations:** ^1^State Key Laboratory of Desert and Oasis Ecology, Xinjiang Institute of Ecology and Geography, Chinese Academy of Sciences, Urumqi, China; ^2^Research Center for Ecology and Environment of Central Asia, Chinese Academy of Sciences, Urumqi, China; ^3^University of Chinese Academy of Sciences, Beijing, China

**Keywords:** climate change, meteorological factor, nutrient addition, above-ground biomass (AGB), Central Asia

## Abstract

Grasslands provide key resource for the millions of people who are dependent on livestock in Tajikistan. Productivity and species richness (SR) are important characteristics of grassland ecosystems and are greatly affected by nutrient inputs. The effect that climate change might have on these characteristics remains unclear. Here, an *in situ* nitrogen (N) and phosphorus (P) fertilization experiment was conducted at four sites along with an elevational gradient (650, 1,100, 1,250, and 2,000 m) in western Tajikistan over 2 years (2018 and 2019) to examine the influences of nutrient availability and climate change on aboveground biomass (AGB) and SR; precipitation and temperature were also considered to analyze the responses. It demonstrated that enrichment with N, P, and their combinations significantly increased AGB along with an elevational gradient (*p* < 0.05). AGB increased as the concentrations of nutrients added increased. The maximum AGB, which was 2-fold higher compared with control, was observed when 90 kg N ha^–1^year^–1^ and 30 kg P ha^–1^year^–1^ were added. In addition, nitrogen addition alone stimulated greater AGB than P addition, although no significant difference was observed between these two treatments. Enrichment with N, P, and their combination had no significant effect on SR; however, SR significantly changed at different elevation. Elevation had direct effect on precipitation and temperature, which, in turn, resulted in variation in AGB and SR. Moreover, both nutrient and elevation had significant effect on AGB and SR, but there was no interaction effect of them. AGB and SR interacted with significant negative correlation. In the high-elevation area, plants grew better in the warmer year (2018); this indicates that grasslands in high mountain areas in Tajikistan might have higher productivity as the climate warms, which will positively affect the economic development of the country.

## Introduction

Grasslands are important terrestrial ecosystems, accounting for almost 30% of the land area of earth (Bi et al., 2020). They are important providers of various ecosystem services including the production of livestock forage and biodiversity conservation. Currently, grassland ecosystems have experienced severe changes, for example, the decline of grassland productivity and the loss of species diversity, as both climate change and anthropogenic activities have contributed to dynamic variations in these ecosystems (Jiang et al., 2015; Su et al., 2021). Plant productivity and species richness (SR) are both fundamental properties of grassland and those two properties can reflect the structure and function of grassland ecosystems (Tilman et al., 2006; Wu et al., 2020). However, the degree to which these two properties might change under climate change remains unclear.

Nitrogen (N) and phosphorus (P) play vital roles in plant development and are the most important limiting nutrients in various grasslands (Fay et al., 2015). Climate change and human activities have greatly exacerbated grassland degradation. Fertilization is the primary means for improving the productivity of grassland systems (Ineichen et al., 2020). The effect of nitrogen and phosphorus application on the productivity and diversity of grassland ecosystems has been examined by previous studies and the results of these studies are variable (Alhamad et al., 2012; Harpole et al., 2017; Soons et al., 2017). Increase in nitrogen is known to significantly increase the above-ground biomass (AGB) and reduce the species diversity (Zhao et al., 2019; Gao et al., 2021). However, some studies have shown that although nitrogen addition reduced the species diversity, it increased the functional richness (Niu et al., 2014; Mao et al., 2017). Enrichment with phosphorus fertilizer has been shown to have either increased or no effect on ecosystem productivity and species diversity (Menge and Field, 2007; Gao et al., 2021). The effect of phosphorus fertilizer on ecosystems can also be regulated by nitrogen fertilizer (Bracken et al., 2015).

Tajikistan is a mountainous country located in the region of Central Asia, where the grasslands are widely distributed. The main grassland distribution ranges about from 300 to 4,000 m, resulting in different microclimate and grassland types (Zhang et al., 2014). Grasslands are an important resource supporting the economy of Tajikistan. However, the rapid increase in the human population during the last three decades has greatly increased the demand for dairy products and meat; consequently, the overgrazing and overuse of grasslands have resulted in serious grassland degradation (Han et al., 2016). As fertilization is commonly used to improve grassland quality (Ineichen et al., 2020), understanding how fertilization affects grassland productivity and diversity is essential for managing grassland and ensuring the sustainable development and utilization of grassland resources. Meanwhile, the vegetation in this arid area is sensitive to variation in climate, as changes in vegetation in this region are correlated with annual precipitation and temperature fluctuation (Yin et al., 2016). Therefore, in this study, we explored the impacts of nutrient (N and P) addition along with an elevational gradient in western Tajikistan on aboveground biomass and SR in 2018 and 2019. Furthermore, precipitation and temperature were also considered to analyze the responses.

## Materials and Methods

### Study Area Description

**FIGURE 1:**
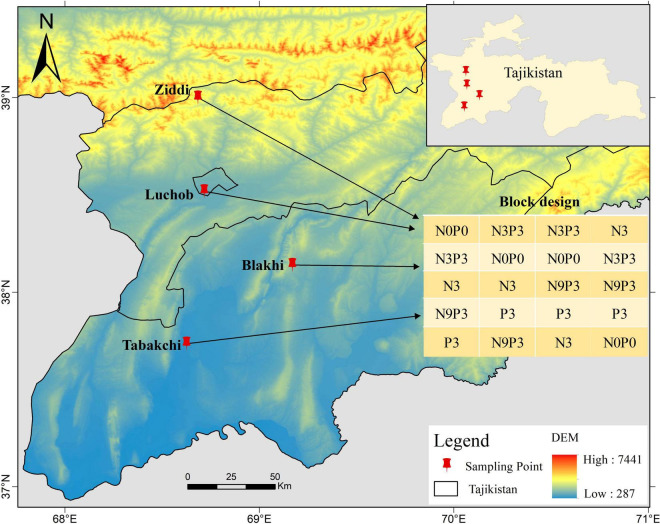
Map of the study area and the experimental design. N0P0–the control; N3—addition of 30 kg N ha^−1^year^−1^, P3—addition of 30 kg P ha^−1^year^−1^, N3P3—addition of 30 kg N ha^−1^year^−1^ and 30 kg P ha^−1^year^−1^, N9P3—addition of 90 kg N ha^−1^year^−1^ and 30 kg P ha^−1^year^−1^. The fertilizer was added *via* a single application. The Shuttle Radar Topographic Mission (SRTM) 30 m digital elevation model (DEM) dataset was obtained from USGS (https://glovis.usgs.gov/).

**FIGURE 2:**
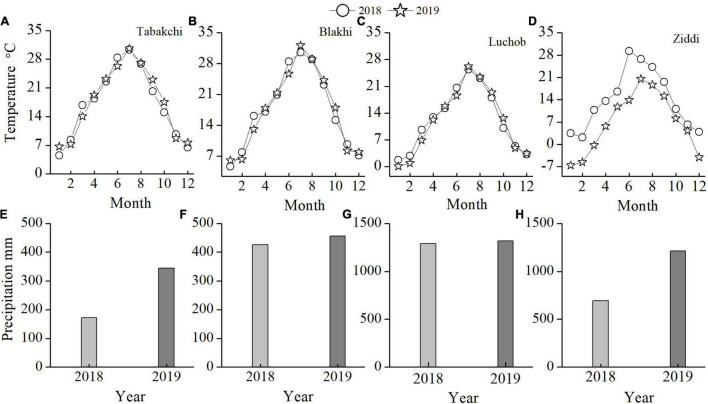
The average monthly temperature **(A–G)** and monthly precipitation **(E–H)** in the four experimental sites.

This study was conducted in Tajikistan (total area: 1.431 × 10^3^ km^2^), which covers a latitudinal range of 36°40′ to 41°04′N and a longitudinal range of 67°22′ to 75°13′E (Figure 1). Tajikistan is known as “the country of mountains” and its mountainous area accounts for about 93% of the total land area (Hafiz, 2008). The area of natural grassland in Tajikistan is 3.6895 million hm^2^ and the distribution of plant communities shows obvious zonal characteristics (Zhang et al., 2014). Depending on vegetation composition, altitude, and utilization, Tajikistan has more than 10 pasture types, which are important resources for the economic development (Ahmadov, 2012). This study was conducted at four sites varying in elevation. The study area experiences a continental climate. The patterns of temperature change at the four sites are similar (Figure 2). The annual average temperature increases from January, peaks in July, and then decreases. The distribution of precipitation is similar among the four experimental sites (Figures 2E–H). Most precipitation is concentrated in winter and spring; summer and autumn are the dry seasons. In Table 1, the annual total precipitation increased with elevation: 172.6 mm in Tabakchi (650 m) in 2018 compared with 1,290.00 and 692.9 mm in Luchob (1,250 m) and Ziddi (2,000 m), respectively.

**TABLE 1:** The location of the four experimental sites and the total precipitation and annual average temperature among the four sites.

Site	Latitude	Longitude	Altitude (m)	TP (mm)		AAT (°C)	
				2018	2019	2018	2019
Tabakchi	37°51.55′°N	68°57.56′°E	650	172.60	344.50	17.28	17.60
Blakhi	38°14.05′°N	69°17.02′°E	1,100	426.30	456.70	17.51	17.51
Luchob	38°39.91′°N	68°39.38′°E	1,250	1,290.00	1,317.80	12.35	12.05
Ziddi	39°02.19′°N	68°49.37′°E	2,000	692.90	1,211.90	13.96	6.77

*TP, total precipitation; AAT, annual average temperature.*

### Experimental Design

Our *in situ* field experiments were conducted in West Tajikistan from 2018 to 2019. Experiments were conducted at four sites (Figure 1) along with an elevational gradient: Tabakchi (650 m), Blakhi (1,110 m), Luchob (1,250 m), and Ziddi site (2,000 m) to examine the effect of nutrient (N and P) addition on the productivity and the SR of pasture ecosystems. At each site, four blocks (20 m × 3 m) were setup at each site with a 1-m wide buffer zone between the blocks. Each block was further divided into 20 plots for different nutrition treatment (Figure 1). The size of each plot was 3 m × 3 m with 1 m wide buffer zones between the plots. Five nutrition treatments were conducted in each block and the four replicates of each five treatments were randomly assigned: CK—control; N3—addition 30 kg Nha^–1^ year^–1^, P3—addition 30 kg Pha^–1^ year^–1^, N3P3—addition 30 kg Nha^–1^ year^–1^ and 30 kg Pha^–1^ year^–1^, and N9P3—addition 90 kg Nha^–1^ year^–1^ and 30 kg Pha^–1^ year^–1^. Nutrient addition was added *via* a single application after snow melting. Stainless steel sheets were used to prevent water infiltration between treatments, 40 cm of the sheets were inserted into the ground and 10 cm of the sheets were left above ground. The meteorological data were obtained from the metrological station near the experimental sites.

### Plant Sampling

In 2018 and 2019, the name and number of plant species were recorded in each 1 m × 1 m quadrats under different nutrient treatments along with an elevational gradient each year during the peak of the growing season. Above-ground plants were harvested at the end of the growth period in 1 m × 1 m quadrats. AGB was determined by oven drying at 68°C until a constant weight was achieved. Total SR was recorded by noting all the plant species present within 1 m × 1 m quadrats.

### Statistical Analysis

A linear mixed-effects models were used to analyze the effect of different nutrition addition treatments on plant AGB and SR along with an elevational gradient, with nutrient and elevation as independent variable and years as a random factor. Structural equation modeling (SEM) was used to evaluate and quantify the effects of elevation, precipitation, temperature, and nutrient addition on plant AGB. The SEM was fit using the “lavaan” package in R version 3.3.2 (R Core Team, 2016). The model was considered to be a good fit based on the following criteria: an insignificant (*p* > 0.05) chi-square test statistic, chi-square/df < 3, root mean square error of approximation (RMSEA) < 0.05, goodness of fit index (GFI) > 0.95, and standardized root mean square residual (SRMR) < 0.09 (Liu et al., 2020). The Tukey’s *post hoc* test following the one-way ANOVA was used to compare the differences of mean values at *p* < 0.05. All the statistical analyses were conducted in R version 3.3.2 (R Core Team, 2016). Origin 2015 software (Origin Lab, Northampton, MA, United States) was used to prepare figures.

## Results

### Above-Ground Biomass and Species Richness Under Different Nutrition Addition Treatment Along With an Elevational Gradient

**FIGURE 3:**
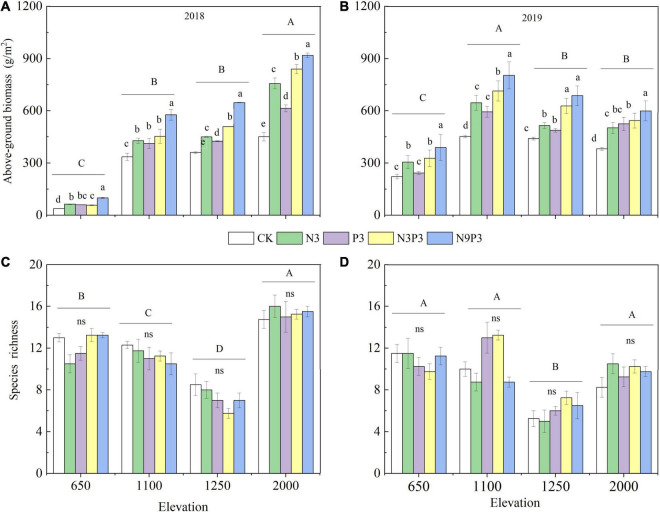
Changes in the above-ground biomass **(A,B)** and species richness **(C,D)** changing under different nutrient treatments at different elevations in 2018 (left) and 2019 (right). Within each panel, groups with the same capital letters above them are not significantly different at *p* < 0.05 (Duncan’s test). Within each group of five bars, bars sharing the different letters are significantly different from each other at *p* < 0.05. Letters are not presented when none of the five bars differs significantly from any of the others (Duncan’s test), ns means that differences are not significant. The data were showed as mean ± SE.

In both 2018 and 2019, the addition of nutrients greatly increased AGB as the elevation increased (Figures 3A,B and Table 2; *p* < 0.05). AGB was higher under N addition than under P addition; however, no significant differences were observed between these treatments. AGB was higher under nutrient addition compared with the control and the maximum AGB was observed under N9P3. Nutrient addition significantly increased AGB 2-fold at Tabakchi and Ziddi relative to the control; AGB at these sites ranged from 37.65 to 99.50 g/m^2^ and from 451.30 to 918.86 g/m^2^, respectively. The effect of nutrient addition on species diversity did not significantly differ at these two sites. However, elevation had a significant effect on species diversity (Figures 3C,D and Table 2). SR was lowest at Luchob (6 species/m^2^) (Figures 3C,D). There was no interaction effect between nutrients and elevations and they independently affected AGB and SR (Table 2).

**TABLE 2:** Summary of the linear mixed-effects models relating fixed factor (N, E) and random factor (Y) for the grassland above-ground biomass and species richness.

Source	AGB		SR	
	*F*	*P*	*F*	*P*
N	14.971	0.000***	0.2684	0.898
E	156.448	0.000***	125.5307	0.000***
N × E	1.281	0.237	1.071	0.389

N means different nutrient addition and E means different elevation, N × E means the interaction. ***Significant difference at p < 0.001 level.

### Influence of Elevation P and T on Aboveground Biomass and Species Richness

**FIGURE 4:**
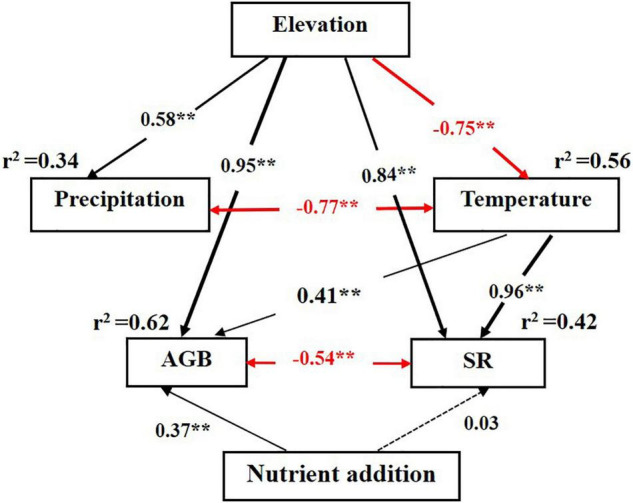
Structural equation model for the effect of environmental factors and nutrient inputs on the plant above-ground biomass. Red and black arrows indicate positive and negative relationships, respectively. Single-headed arrows represent causal relationships and two-headed arrows indicate correlation. Dotted arrows represent non-significant paths (*p* > 0.05). Values adjacent to arrows indicate standardized path coefficients. The proportion of variance explained (*r*^2^) appears alongside response variable in the model. Significance levels are denoted with **p < 0.01.

**FIGURE 5:**
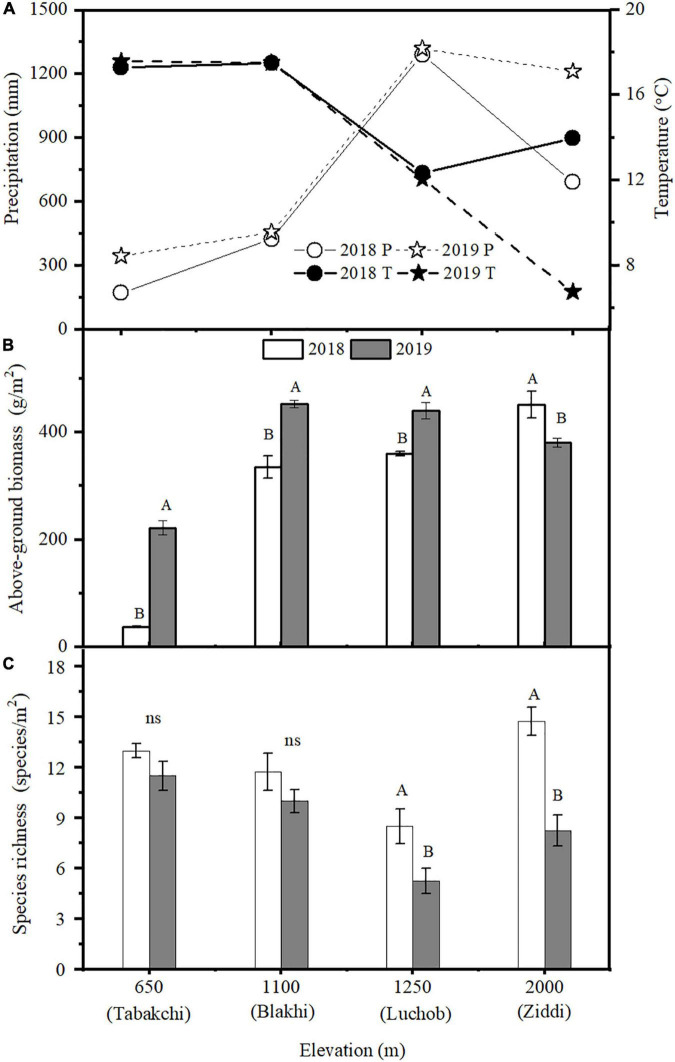
Annual total precipitation and annual average temperature at different elevations **(A)**, the average above-ground biomass **(B)**, and species richness **(C)** in 2018 and 2019. Data of the control treatment were used (mean ± SE). Different capital letters indicate significant difference between years at the same elevation (*p* < 0.05), ns, no significant difference.

The SEM analysis showed that elevation had significant (*p* < 0.01) positive direct and indirect effects on plant AGB and SR. Elevation had direct effects on annual total precipitation and annual average temperature, but positive direct effect on precipitation and negative direct effect on temperature. Annual total precipitation and annual average temperature were significant negatively correlated (*p* < 0.01). Nutrient addition had positive direct effect on plant AGB, but nutrient addition had no significant direct effect on SR (*p* > 0.05). AGB and SR had negative correlation (Figure 4). AGB was observed higher in 2018 (more precipitation) at lower and middle elevation areas (Figures 5A,B). Contrary to expectations, it showed the opposite result at the high elevation (Ziddi). In the warm year (2018), AGB at higher elevation was more than in the cold year (2019) though with less precipitation (Figures 5A,B). SR had no significant between years at Tabakchi and Blakhi. However, SR had significant difference between years at Luchob and Ziddi with higher SR in 2018 (Figure 5C).

## Discussion

### Effect of Nutrients on the Grass Aboveground Biomass and Species Richness

Nitrogen and phosphorus are two limiting nutrients that regulate the development of grassland ecosystems (Fay et al., 2015). Nitrogen and phosphorus addition significantly increased the AGB as the elevation increased and AGB increased as the concentrations of nutrients applied to the soil increased. These results are consistent with previous studies in grassland ecosystems showing that nitrogen and phosphorus additions stimulated primary production (Li et al., 2015; Zhao et al., 2019). Moreover, our findings indicated that nutrient addition was more effective at promoting plant growth compared to the control. The effect was enhanced by the increased precipitation at the middle to lower elevations (Tabqichi, Blakhi, and Luchob). The AGB was higher in 2019 than in 2018 in the nutrient addition treatments because precipitation was higher in 2019 compared with 2018 (Figures 3A,B and Table 1). Other studies have shown that increase in water promotes nutrient availability and increases primary productivity (Yang et al., 2011; Fan et al., 2013). A global scale experiment of nutrient addition in grasslands has shown that N addition increases biomass to a greater degree than P addition. The addition of both N and P was more beneficial for biomass increasing than adding only one of these two elements (Fay et al., 2015). It is in an agreement with our 2-year nutrient fertilizer experiment. The amount of the AGB with adding only nitrogen was higher than that adding only phosphorus. Combined NP enrichment was more beneficial for the plant growth than either along nitrogen or phosphorus addition. Furthermore, the AGB showed a positive response to nutrient concentration, especially to the nitrogen. These results indicated that nitrogen is more likely to be the limiting nutrient for biomass production in Tajikistan grassland as the elevation increases and the effect of phosphorus addition on the plant growth was regulated by nitrogen nutrient.

Species diversity plays an important role in maintaining the structure of ecosystems (Hautier et al., 2020). Increase in nitrogen is thought to significantly increase the AGB and reduces the species diversity (Humbert et al., 2016). Several meta-analyses have shown that nitrogen enrichment generally reduced plant SR in terrestrial ecosystems, especially in grassland ecosystems (Soons et al., 2017; Yue et al., 2020). However, the results of this study were not fully consistent with the results of these previous studies. Nitrogen, phosphorus, or their combinations addition did not increase or decrease SR, but SR exhibited significant responses within elevations. Among with an elevational gradient, SR showed significant different with Lucholb that had the lowest SR. The reason for that might be explained by short-term nutrient addition. Long-term experiments may be necessary for characterizing the effect of nutrient addition on species diversity. The previous study found that the greater increase in plant productivity with long-term nitrogen addition, the greater decline in plant diversity, and vice versa (Yue et al., 2020). This study conducted nutrient addition for 2 years; the SR to nutrient availability may be not so sensitive, which needs to take longer time fertilizer experiment to analyze the response. Overall, nutrient limitation often increases with increasing elevation (Johnson et al., 2000), but some recent observational studies suggested that the relative importance of different limiting nutrients changes with elevation (Fisher et al., 2013; Sundqvist et al., 2014). In this study, though both nutrient and elevation had significant effect on the AGB or SR, there was no interaction effect of them on the AGB or SR and they independently affected AGB and SR (Table 2).

### Changes in Biomass and Diversity Driven by Environmental Variables

Precipitation and temperature are considered to be the major limiting factors in arid and semiarid region and high latitude ecosystems and these systems are, therefore, predicted to be particularly sensitive to climate change (IPCC, 2014). Most previous studies have found productivity to be linearly correlated with mean annual precipitation (Hu et al., 2007; Mowll et al., 2015), as the amount of precipitation largely determines the productivity of the grassland ecosystem (Esteban and Osvaldo, 2000; Bai et al., 2004). Other studies indicated that declining temperatures associated with increasing elevation can directly influence ecosystem properties (Bahram et al., 2012; Sundqvist et al., 2014). Our study area spans different elevations, so there are differences in rainfall and temperature. Through the SEM analysis, we can know that annual total precipitation and annual average temperature variation were directly significantly affected *via* elevational gradient, with positive on precipitation and negative on temperature. Meantime, precipitation and temperature were negatively correlated (Figure 4). Therefore, consistent with previous studies, the AGB and SR were affected not only by elevation, but also by annual total precipitation and annual average temperature (Figure 5). Different effects of precipitation and temperature can be observed at different elevations correlated with the vegetation. In this study, vegetation changed from desert grassland to mountain grassland from the south to the north experimental site and correspondingly, the AGB increased. Specifically, the AGB in desert grassland was more sensitive to variation of precipitation, which can be more than quadrupled in 2019 (the year with more precipitation) compared with 2018. Besides precipitation, annual average temperature also had a significant positive relationship with the grassland AGB. Generally, warming had a negative indirect effect on plant productivity because of increased soil drought associated with higher evapotranspiration and temperature (Dulamsuren et al., 2013). Nevertheless, in our high-elevation experimental site Ziddi, the year (2018) with higher temperature had the higher AGB compared with the year with relative lower temperature (2019). This result indicated that warming might be beneficial for the plant growth. Since global warming is an indisputable fact (IPCC, 2014), the grassland in high mountain areas in Tajikistan may have higher productivity in the future based on current warming trends and this would have a positive effect on the economic development of Tajikistan.

Productivity and diversity are two major properties of ecosystems and environmental factors can affect the productivity; the variation will lead to the change of diversity simultaneously and vice versa (Gross and Cardinale, 2007; Wang et al., 2019). In arid areas, water largely determines the plant growth, thus competition among plants is mainly access to water resources (Mu et al., 2021). When precipitation increases, the rapid growth of some species will further lead to the competition for light, which may lead to the decrease in diversity. Therefore, the relationship between growth ability and species diversity is mainly caused by the competition of resources (Miki, 2009). The competition for water and light, thus, provides the best explanation of our finding.

## Conclusion

The AGB and SR in this mountainous arid community were largely affected by nutrient availability and elevation. Nutrient enrichment was beneficial for the plant growth. The AGB increased with the more nutrient concentration along with an elevational gradients and the maximum increment of the AGB can be quadrupled compared with CK. Nitrogen adding alone was more beneficial for accumulation of the AGB compared with phosphorus adding. Enrichment with N, P, and their combination had no significant effect on SR; however, SR significantly changed at different elevation. Moreover, both nutrient and elevation had significant effect on the AGB and SR, but there was no interaction effect of them. The AGB and SR interacted with significant negative correlation. Elevation had direct effect on precipitation and temperature, which, in turn, caused variation in the AGB and SR.

## Data Availability Statement

The original contributions presented in the study are included in the article/supplementary material, further inquiries can be directed to the corresponding authors.

## Author Contributions

Y-ML and J-FM conceived the study. L-LF conducted the experiments, analyzed the data, and drafted the manuscript. OM, K-HL, and X-XM performed the experiments. All authors showed in the article contributed to study conceptualization, read, and approved the final version of this manuscript.

## Conflict of Interest

The authors declare that the research was conducted in the absence of any commercial or financial relationships that could be construed as a potential conflict of interest.

## Publisher’s Note

All claims expressed in this article are solely those of the authors and do not necessarily represent those of their affiliated organizations, or those of the publisher, the editors and the reviewers. Any product that may be evaluated in this article, or claim that may be made by its manufacturer, is not guaranteed or endorsed by the publisher.
